# Supplementary material for: Membrane-Associated, Not Cytoplasmic or Nuclear, FGFR1 Induces Neuronal Differentiation
Source: Cells. 2019 Mar 14;8(3):243. doi: 10.3390/cells8030243 (PMC6468866; doi:10.3390/cells8030243)
Supplement: Supplementary file 1 [file cells-08-00243-s001.pdf]

Article

Membrane-Associated, Not Cytoplasmic or Nuclear, FGFR1 Induces Neuronal Differentiation

Katalin Csanaky [1], Michael W. Hess [2] and Lars Klimaschewski [1,*]

[1] Division of Neuroanatomy, Medical University of Innsbruck, 6020 Innsbruck, Austria; katalin.csanaky@i-med.ac.at

[2] Division of Histology and Embryology, Medical University of Innsbruck, 6020 Innsbruck, Austria; michael.hess@i-med.ac.at

* Correspondence: lars.klimaschewski@i-med.ac.at; Tel.: +43-512-9003-71160

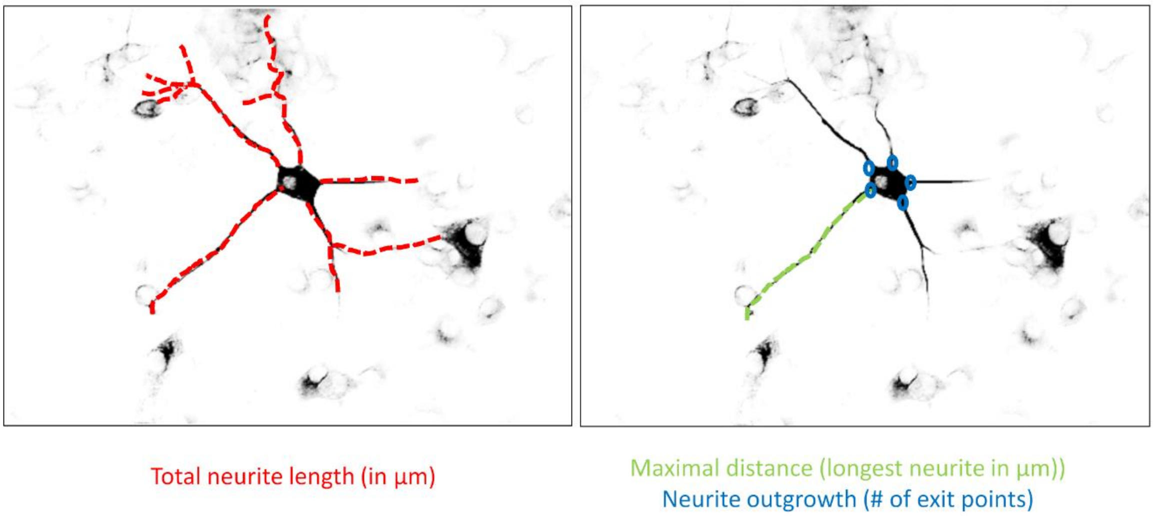

Figure S1. PC12 cells were defined as differentiated if they exhibited at least one process of more than one cell body diameter in length. Total neurite length (sum of the length of all neurites extending from the cell body; red), the maximal distance (length of longest neurite; green) and the overall neurite outgrowth were calculated (number of neurites/cell; blue). Each experiment was conducted in triplicate.

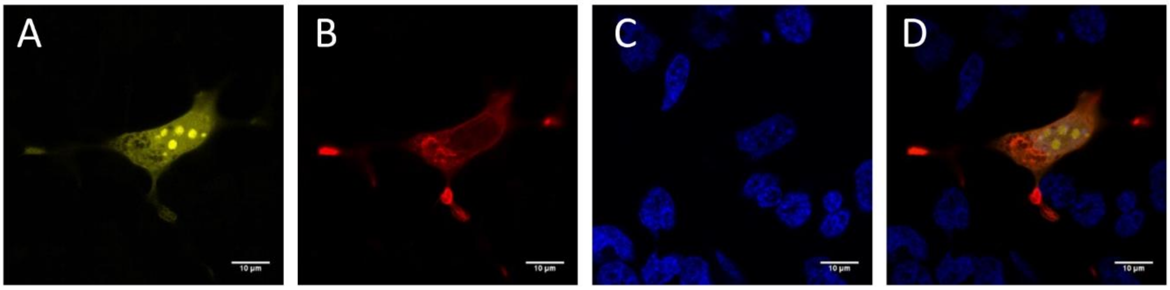

Figure S2. Immunofluorescence microscopy of HEK293 cells co-transfected with mV-nucl-opto-FGFR1s and LifeAct-mCherry. (**A**) mV-nucl-opto-FGFR1 is located in the nucleus (enrichment in nuclear speckle domains) and treatment with 25 µM importazole results in additional, diffuse cytoplasmic yellow fluorescence. (**B**) LifeAct-mCherry for visualization of the filamentous actin cytoskeleton. (**C**) Fixed cell nuclei are stained with Hoechst (blue) and (**D**) represents overlay. Scale bars = 10 µm.

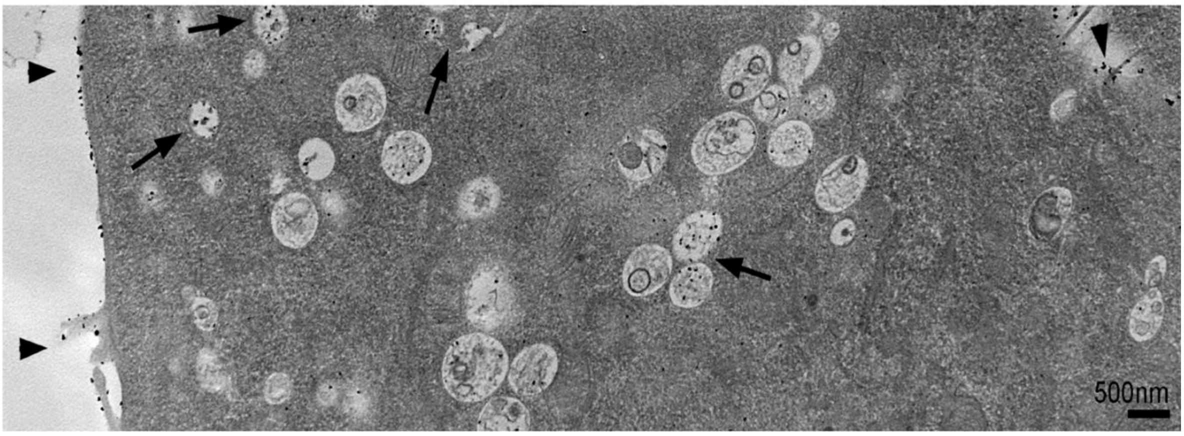

Figure S3. Pre-embedding immunogold electron microscopy of high-pressure frozen U251 glioblastoma cells reveals mV-mem-opto-FGFR1 at the plasma membrane (arrowheads) and in endosomes/lysosomes (arrows). Scale bar = 500 nm.

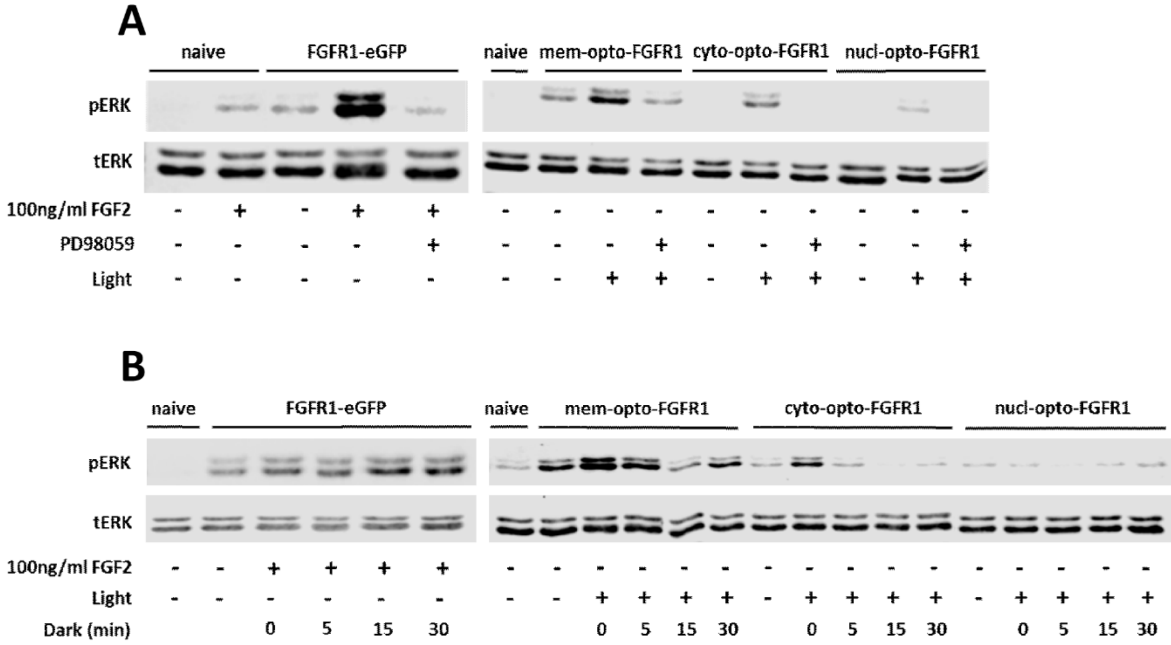

Figure S4. Phosphorylation of ERK in response to ligand and blue light stimulation in HEK293 cells expressing FGFR1-eGFP or opto-FGFR1s. (**A**) Induction of ERK was blocked by PD98059 treatment. (**B**) Blue light stimulation for 5 min following mem-opto-FGFR1 or cyto-opto-FGFR1 transfection significantly elevated pERK levels which was reduced to pre-stimulation levels within 15 min. In contrast to mem-opto-FGFR1, light-induced ERK activation decreased shortly after cessation of the light stimulus in cyto-opto-FGFR1 transfected cells.

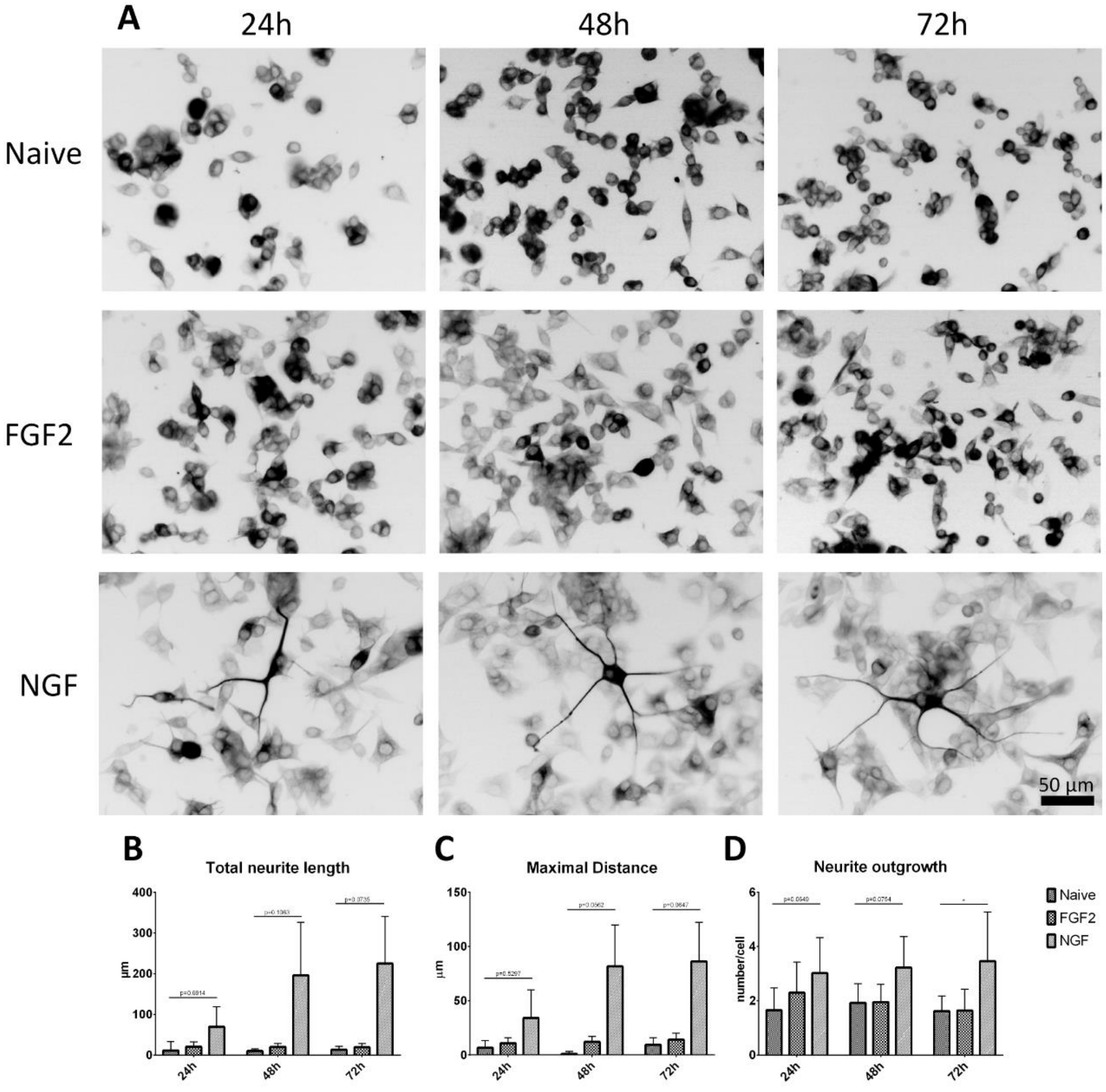

E

	Naive PC12
**FGFR1**	32.1 ± 0.1
**FGFR2**	31.8 ± 0.5
**FGFR3**	27.7 ± 0.2
**FGFR4**	31.7 ± 0.4
**Actin**	17.7 ± 0.2

Figure S5. (**A**) Cells were treated with 100 ng/mL NGF or 100 ng/mL FGF2 + 1 µg/mL HepS for 24, 48 and 72 hours and stained for neuron-specific class III β-tubulin to identify neurites. Scale bar indicates 50 µm. (**B**–**D**) Quantification of morphological parameters (total neurite outgrowth, longest process and number of processes per cell; means ± SD, 30 < n < 50, *$p < 0.05$). For statistical analysis two-way ANOVA with Tukey test was used. (E) Mean threshold cycles of qRT-PCR measurements of FGFR1-4 mRNA (± SD).

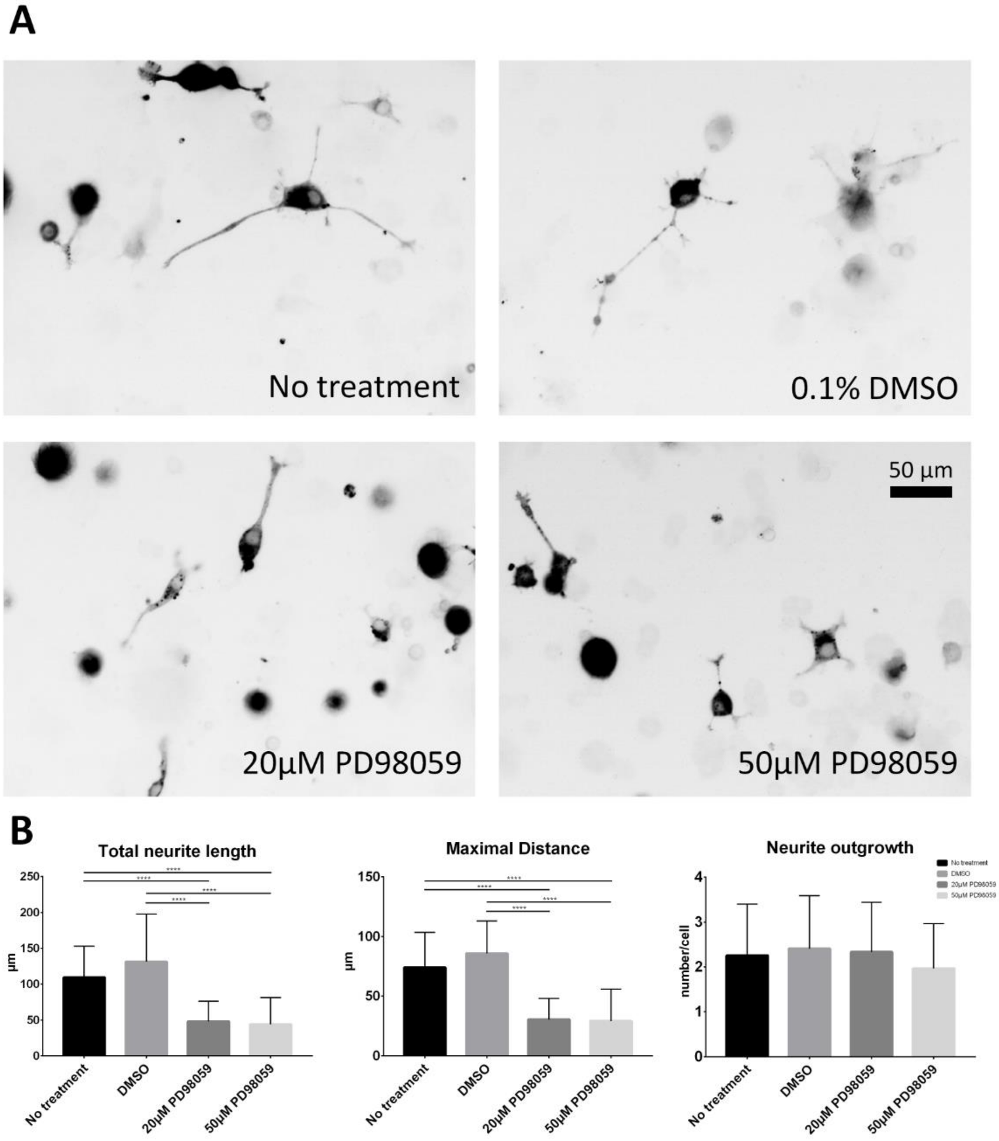

Figure S6. Inhibition of blue light induced PC12 cell differentiation by ERK blockade. (**A**) PC12 cells were transiently transfected with 1 µg mV-mem-opto-FGFR1. After serum starvation, cells were pre-treated with 20 µM or 50 µM MEK/ERK inhibitor (PD98059) for 2 hours and stimulated with intermittent (5 min ON/55 min OFF) 2.5 $\mu W/mm^2$ blue light for 48 hours followed by immunostaining for neuron-specific class III β-tubulin. Scale bar indicates 50 µm. (**B**) Quantification of morphological parameters (total neurite outgrowth, longest process and number of processes per cell; mean ± SD, $15 < n < 35$, $^{****}p < 0.001$).
